# Radiolucent cerclage for humerus fractures: beware of radial nerve injury—a case report

**DOI:** 10.1093/jscr/rjae370

**Published:** 2024-05-30

**Authors:** Maria Dadabhoy, Rory Cuthbert, Kapil Sugand, Anuhya Vusirikala, Michael Fox, Anna Panagiotidou, Marco Sinisi, Tom Quick

**Affiliations:** Peripheral Nerve Injury Unit, Royal National Orthopaedic Hospital, Stanmore HA7 4LP, United Kingdom; Peripheral Nerve Injury Unit, Royal National Orthopaedic Hospital, Stanmore HA7 4LP, United Kingdom; Peripheral Nerve Injury Unit, Royal National Orthopaedic Hospital, Stanmore HA7 4LP, United Kingdom; Department of Surgery and Cancer & MSk Lab, Imperial College, London W12 0BZ, United Kingdom; Peripheral Nerve Injury Unit, Royal National Orthopaedic Hospital, Stanmore HA7 4LP, United Kingdom; Peripheral Nerve Injury Unit, Royal National Orthopaedic Hospital, Stanmore HA7 4LP, United Kingdom; Peripheral Nerve Injury Unit, Royal National Orthopaedic Hospital, Stanmore HA7 4LP, United Kingdom; Peripheral Nerve Injury Unit, Royal National Orthopaedic Hospital, Stanmore HA7 4LP, United Kingdom; Peripheral Nerve Injury Unit, Royal National Orthopaedic Hospital, Stanmore HA7 4LP, United Kingdom; Centre for Nerve Engineering, University College London, London WC1N 1AX, United Kingdom

**Keywords:** radial nerve, humeral fractures, peripheral nerve injuries

## Abstract

A 73-year-old woman was referred to a National Centre for Peripheral Nerve Injury with a post-operative left radial nerve degenerative lesion following open reduction and internal fixation of a proximal third humerus fracture using radiolucent Arthrex FiberTape® Cerclage as an adjunct to plating to improve stability. Intra-operative photographs illustrate compression of the radial nerve under the cerclage construct. Use of radiolucent cerclage for humerus fractures is increasing with modern systems capable of withstanding an ultimate load of 4300 N. We highlight the risk of debilitating neurological injury when not deployed safely and describe anatomical high-risk zones for injury. We emphasize the impact of delay in diagnosis and treatment.

## Introduction

Use of radiolucent cerclage for humerus fractures is increasing. The Arthrex FiberTape® Cerclage system provides an all-suture alternative to metal cables for circumferential fracture fixation. A disposable tensioner is used to provide 80 N of force with each cycle, with the construct capable of withstanding up to 4300 N [1]. FiberTape® is radiolucent, providing the advantage of reducing radiographic interference but also meaning structures can be trapped under the cerclage without radiographic evidence.

The radial nerve is at risk at any point in its course around the humerus and can be entrapped on metaphyseal and diaphyseal fixation. The cerclage technique is most commonly deployed within the diaphysis of the humerus. Radial nerve injury commonly presents with wrist drop following deinnervation of the long extensors of the wrist and digits with alteration of sensation and sympathetic function in the distribution of the superficial radial nerve [2].

Injury to a nerve associated with fracture fixation is an important complication to recognize and the British Orthopaedic Association has established guidelines for Peripheral Nerve Injury [3]. The guideline espouses the essential tenet of repeated examination, high index of suspicion, and early specialist referral.

## Case report

A 73-year-old right-handed female was referred to our national Peripheral Nerve Injury unit with a post-operative, left radial nerve lesion confirmed on clinical examination. She had sustained a proximal third humeral fragility fracture following a low-energy fall from standing which was managed non-operatively.

**Figure 1:**
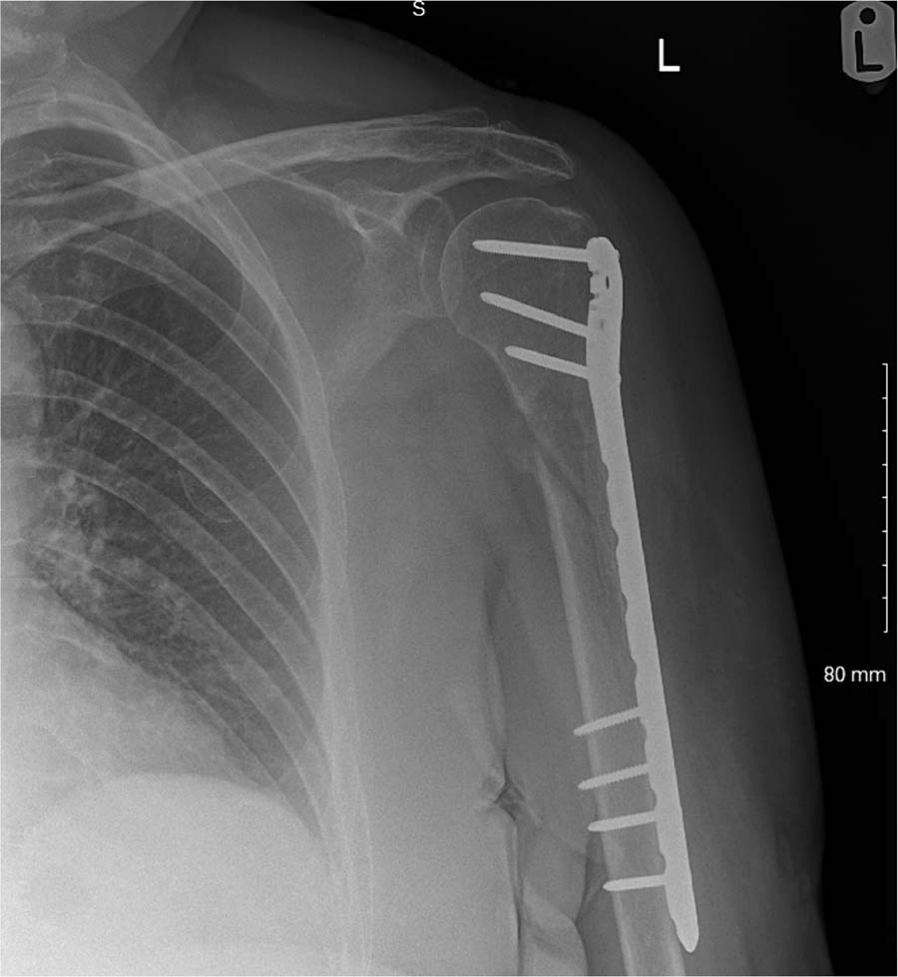
Post-operative radiographs following humerus fixation.

The fracture was neurologically intact at presentation to the local service but at 3 months follow-up, painful hypertrophic non-union was evident so open reduction and internal fixation [with a De Puy Synthes proximal humeral internal locking system (PHILOS®) and two Arthrex FiberTape® Cerclages; Fig. 1] was performed. Immediately post-operatively, the patient had MRC 0/5 power in brachioradialis, extensor carpi radialis longus, extensor digitorum communis, extensor pollicis longus, and extensor indicis proprius with spontaneous paresthesia and anesthesia in the superficial radial nerve cutaneous distribution.

Magnetic resonance imaging conducted 7 weeks post-fixation demonstrated oedema adjacent to the radial nerve but was unequivocal for transection due to metal and inflammatory artifacts. Ultrasound demonstrated an ill-defined hypoechoic area at the site of the fracture ‘which could represent scarring’. Nerve conduction studies confirmed a degenerative radial nerve lesion distal to triceps. When the patient was reviewed in the Peripheral Nerve Injury Unit at 2 months post-op, a decision was made to proceed with urgent exploration and neurolysis.

**Figure 2:**
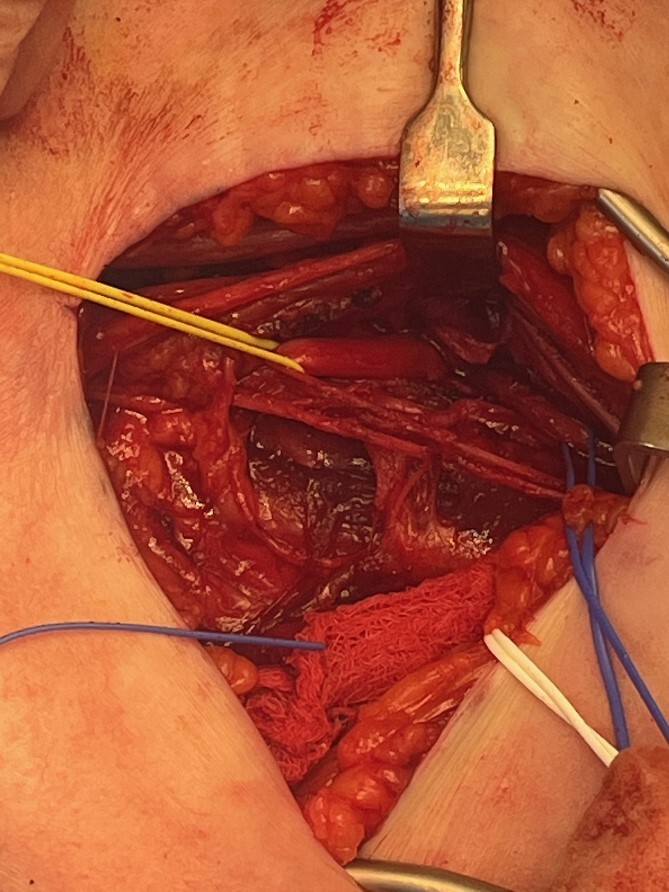
Left radial nerve identified proximally with yellow sling and distally with blue sling showing compression below the Arthrex FiberTape® Cerclage.

**Figure 3:**
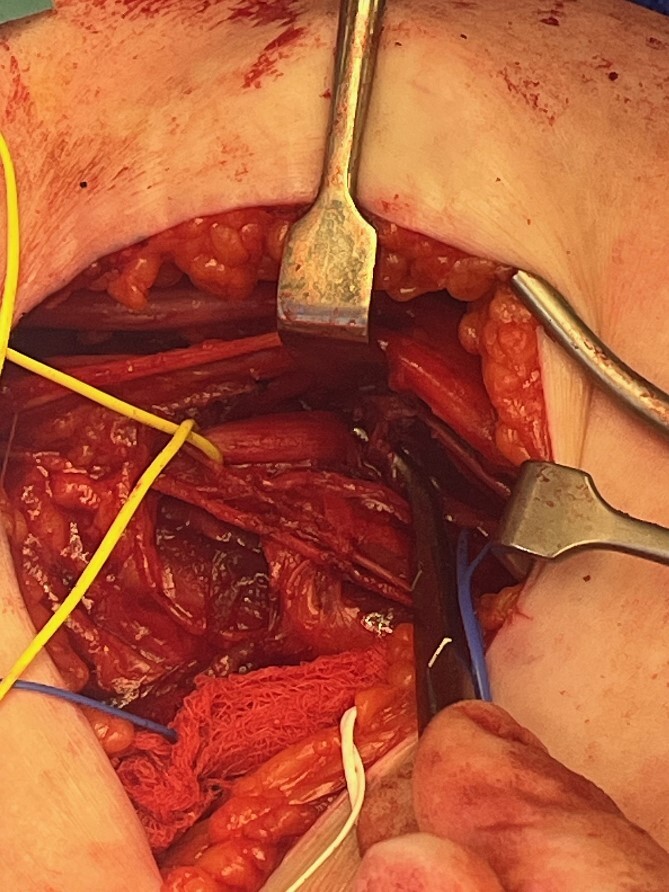
Arthrex FiberTape® Cerclage isolated at site of compression.

**Figure 4:**
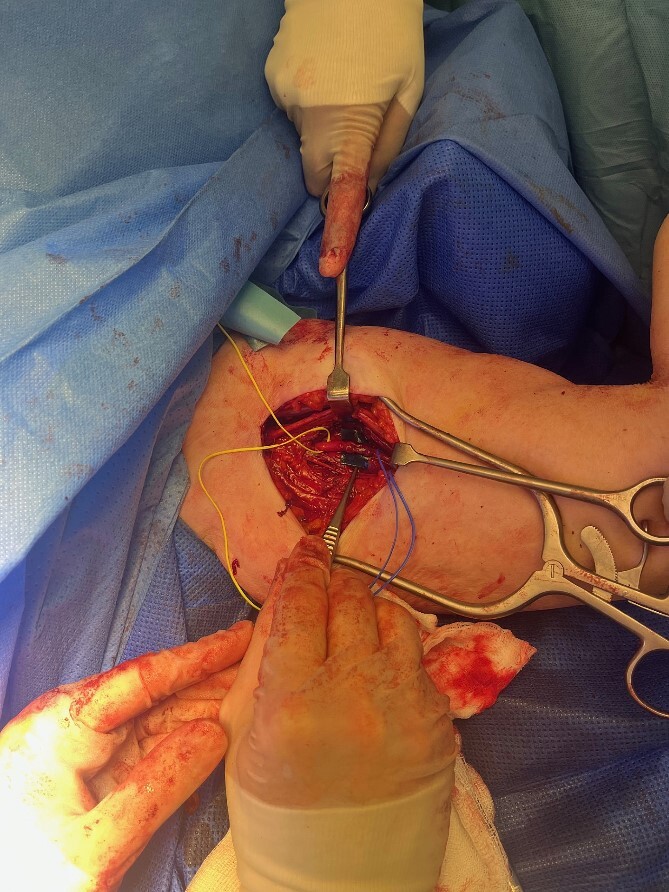
Severe radial nerve axonal injury with epineurial loss and exposed fascicles.

The radial nerve was identified via an anterolateral approach and followed proximally through an axillary approach. The radial nerve was found to be entrapped by the Arthrex FiberTape® Cerclage as it began its descent in the spiral groove (Fig. 2). The nerve was hypochromic and severely crushed by the Arthrex FiberTape® Cerclage with significant epineurial loss leading to exposed fascicles (Fig. 3) over a length of 4 cm either side of the compression point. After neurolysis the Arthrex FiberTape® Cerclage was incised to release the nerve (Fig. 4). The decision at this point was that, due to the length of the lesion (the proximal stump some 30 cm proximal to the lateral epicondyle with a 10 cm lesion), graft reconstruction was not in the patient’s best interest and a review was planned to consent the patient to either tendon transfer or distal nerve transfer reconstruction.

## Discussion

We present a previously unreported case of iatrogenic radial nerve injury following the use of Arthrex FiberTape® Cerclage. There are several important lessons for orthopedic surgeons from this case.

The radial nerve arises in the axilla as one of the terminal branches of the brachial plexus. It traverses through the triangular interval into the posterior compartment of the arm where it supplies the three heads of triceps. The nerve travels deep to the long head of triceps and winds around the spiral groove before piercing the lateral intermuscular septum to enter the anterior compartment [2]. The radial nerve can be injured by fractures of the humerus (~10%) [4] or during its fixation. Safe zones have been identified for use of cerclage constructs in the humerus. The middle third should be utilized with extreme caution due to its proximity to the spiral groove and risk of injury post cerclage in 75% of cases [5, 6]. Studies have measured the radial nerve as being 20.7 ± 1.2 cm proximal to the medial epicondyle to 14.2 ± 0.6 cm proximal to the lateral epicondyle [7]. This distance is further influenced based on the flexion angle of the elbow [8]. Cerclage at the proximal or distal third of the humeral shaft is significantly safer [5]. It must be recognized however that the neurovascular structures running the length of the upper arm are at risk in any cerclage technique and meticulous technique and awareness of neurologic anatomy is important for safe use.

Technical recommendations to avoid injury to the radial nerve include soft tissue release, clearance of fracture hematoma, and mobilization of the fracture to ensure that the cerclage is placed under direct vision [9]. A blunt passing hook adjacent to the bone can ensure that there is no compression of structures beneath the cerclage. It is particularly important to ensure Arthrex FiberTape® is carefully utilized given it cannot be visualized on post-operative radiographs so suspicion for cerclage as a cause of radial nerve injury may not be raised.

All patients undergoing humeral shaft fixation should have a thorough pre- and post-operative assessment including clear documentation of nerve function by the primary surgeon as this is a recognized risk of the surgery as highlighted by the British Orthopaedic Association Guidelines [3]. Immediate advice should be sought from the local Peripheral Nerve Injury network if a new sensorimotor deficit is identified following surgery, especially if there is neuropathic pain or, if in the absence of pain, appropriate conservative measures have not improved symptoms and signs [3]. Surgeons utilizing radiolucent cerclage should be aware of the risks posed and urgently explore the nerve in the event of a new post-operative neurological deficit.

## Conflict of interest statement

The authors declare no conflict of interest—financial or otherwise.

## Funding

There was no funding received for this case report. However, the publication costs have been funded by the Imperial Open Access Fund (Imperial Fund) on behalf of Imperial College London, UK.

## Consent for publication

Informed consent has been obtained from the patient for publication of the case report and the accompanying images. All details are anonymized in keeping with patient confidentiality.
